# Supplementary material for: A VPS33B CRISPR knockout study: In vitro evidence of an adhesion defect
Source: PLoS One. 2026 Feb 13;21(2):e0343240. doi: 10.1371/journal.pone.0343240 (PMC12904430; doi:10.1371/journal.pone.0343240)
Supplement: S1 Table — 20-nucleotide sequences of target-specific sgRNA (single guide RNA) portion of plasmids used for CRISPR knockout of VPS33B (counted from the beginning of the NCBI sequence). (DOCX) [file pone.0343240.s001.docx]

| **Plasmid set** | **Plasmid A sgRNA** | **Plasmid B sgRNA** |
| --- | --- | --- |
| Set 1 | CTTCTCCATGCTGAAGAGGC  nt 414-433> NC_000015.10 | CTCAGGGGCGTCCGGCCGAT  nt 386-405 > NC_000015.10 |
| Set 2 | CTGACTTCTCCATGCTGAAG  nt 410-429 > NC_000015.10 | CGGCCGATGGGGAAAAGCCA  nt 374-393 > NC_000015.10 |
