# Supplementary material for: A VPS33B CRISPR knockout study: In vitro evidence of an adhesion defect
Source: PLoS One. 2026 Feb 13;21(2):e0343240. doi: 10.1371/journal.pone.0343240 (PMC12904430; doi:10.1371/journal.pone.0343240)
Supplement: S2 Table — (DOCX) [file pone.0343240.s002.docx]

| **Gene** | **Forward primer (5’-3’)** | **Reverse primer (5’-3’)** |
| --- | --- | --- |
| *VPS33B* | GAGTCTCTGCGGGGTGTAG  nt 78-96 > GeneID:26276  Chr15: 91022544–91022526 > NC_000015.10 | AGATCCCTAATGGCAAAGATGTC  nt 736-758 > GeneID:26276  Chr15: 91021864–91021886 > NC_000015.10 |
