# Supplementary material for: A VPS33B CRISPR knockout study: In vitro evidence of an adhesion defect
Source: PLoS One. 2026 Feb 13;21(2):e0343240. doi: 10.1371/journal.pone.0343240 (PMC12904430; doi:10.1371/journal.pone.0343240)
Supplement: S3 Table — (DOCX) [file pone.0343240.s003.docx]

| **Gene** | **Forward primer (5’-3’)** | **Reverse primer (5’-3’)** |
| --- | --- | --- |
| Predicted off-target 1 | GCAGGGCCAGATGTTTTCTCTTTG  Chr20: 22233023-22233046 > NC_000020.11 | GCCTGGGCAACAAGAGGGAA  Chr20: 22233297-22233278 > NC_000020.11 |
| Predicted off-target 2 | TAAACTCCAAACGGTGCTGGCAGA  Chr13: 82330877–82330900 > NC_000013.11 | GTTAGAAGGGAAGCTCTGCTGAGG  Chr13: 82331146–82331123 > NC_000013.11 |
| Predicted off-target 3 | GGAATTAGAAGCCTGCTGTCCC  Chr4: 10167688 – 10167709 > NC_000004.12 | CCAGCAGAGATGTCAGGGATAC  Chr4: 10167962 – 10167941 > NC_000004.12 |
| Predicted off-target 4 | GGAATTACAGGCATGAGCGACC  nt 98338 – 98350 > GeneID:118427  Chr1: 101898598 - 101898577 >NC_000001.11 | GGTTGAGCAACTCACCCAACATC  nt 98597 - 98620 > GeneID:118427  Chr1: 101898307 - 101898329 >NC_000001.11 |
