# Supplementary figures and images for: A VPS33B CRISPR knockout study: In vitro evidence of an adhesion defect
Source: PLoS One. 2026 Feb 13;21(2):e0343240. doi: 10.1371/journal.pone.0343240 (PMC12904430; doi:10.1371/journal.pone.0343240)

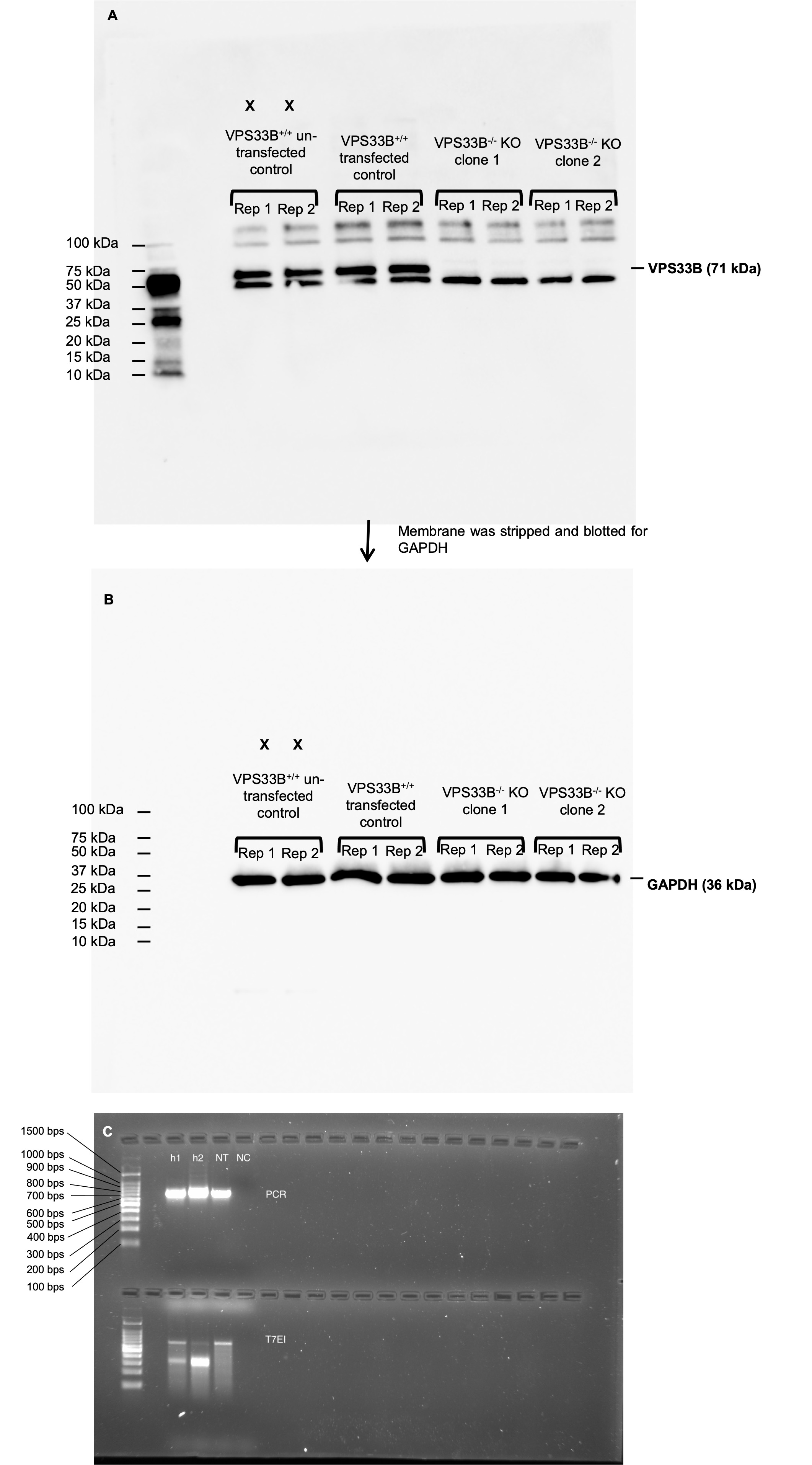

Supplement: S1 Fig — A) VPS33B Western blot displayed in Fig 1B. B) Following VPS33B staining, the membrane was washed and reblotted for GAPDH, also displayed in Figure B. GAPDH intensity was very high, preventing visualisation of the ladder and prior VPS33B stain without severe overexposure. Images were captured with Bio-Rad ChemiDoc™ system. X denotes channels not included in final blot. C) Electrophoresis gel image displayed in Fig 1A acquired using a Gel Doc EZ System. (TIFF) [file pone.0343240.s006.tiff]

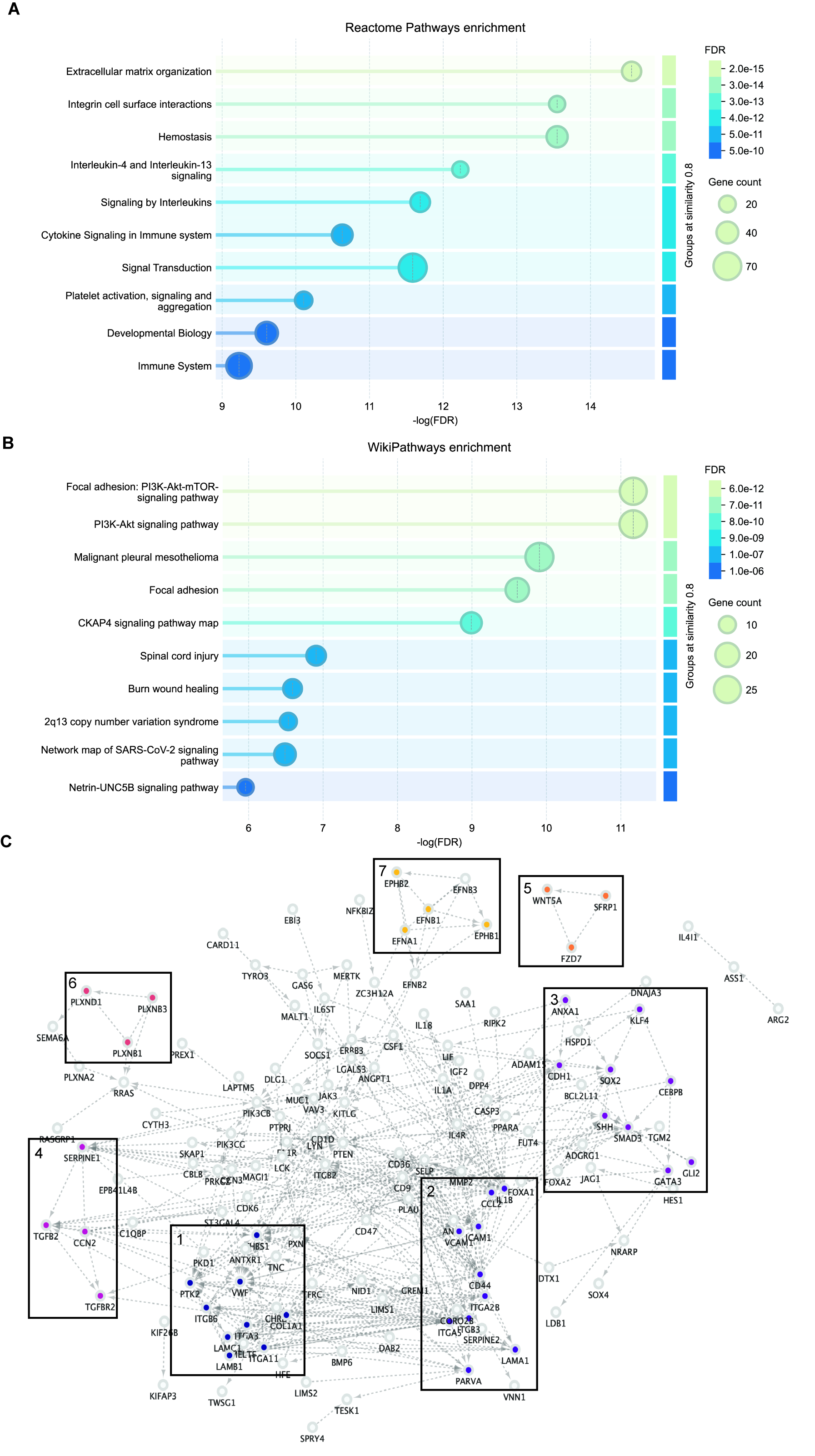

Supplement: S2 Fig — Top 10 A) Reactome Pathway and B) WikiPathways analysis of 209 adhesion-associated DEGs. C) Protein-protein interactions between adhesion-associated DEGs displayed using Cytoscape. PPI modules (highlighted as squares and numbered) were generated with MCODE. Abbreviations: DEG: differentially expressed gene. (TIFF) [file pone.0343240.s007.tiff]
